# The NRTIs Lamivudine, Stavudine and Zidovudine Have Reduced HIV-1 Inhibitory Activity in Astrocytes

**DOI:** 10.1371/journal.pone.0062196

**Published:** 2013-04-16

**Authors:** Lachlan R. Gray, Gilda Tachedjian, Anne M. Ellett, Michael J. Roche, Wan-Jung Cheng, Gilles J. Guillemin, Bruce J. Brew, Stuart G. Turville, Steve L. Wesselingh, Paul R. Gorry, Melissa J. Churchill

**Affiliations:** 1 Centre for Virology, Burnet Institute, Melbourne, Victoria, Australia; 2 Department of Biochemistry and Molecular Biology, Monash University, Victoria, Australia; 3 Department of Microbiology, Monash University, Victoria, Australia; 4 Department of Medicine, Monash University, Victoria, Australia; 5 Department of Infectious Diseases, Monash University, Victoria, Australia; 6 Department of Microbiology and Immunology, University of Melbourne, Melbourne, Victoria, Australia; 7 Department of Neurology, and St. Vincent's Centre for Applied Medical Research, St. Vincent's Hospital, Darlinghurst, New South Wales, Australia; 8 The Kirby Institute, Darlinghurst, New South Wales, Australia; 9 South Australian Health and Medical Research Institute, Adelaide, South Australia, Australia; Centro de Biología Molecular Severo Ochoa (CSIC-UAM), Spain

## Abstract

HIV-1 establishes infection in astrocytes and macroage-lineage cells of the central nervous system (CNS). Certain antiretroviral drugs (ARVs) can penetrate the CNS, and are therefore often used in neurologically active combined antiretroviral therapy (Neuro-cART) regimens, but their relative activity in the different susceptible CNS cell populations is unknown. Here, we determined the HIV-1 inhibitory activity of CNS-penetrating ARVs in astrocytes and macrophage-lineage cells. Primary human fetal astrocytes (PFA) and the SVG human astrocyte cell line were used as *in vitro* models for astrocyte infection, and monocyte-derived macrophages (MDM) were used as an *in vitro* model for infection of macrophage-lineage cells. The CNS-penetrating ARVs tested were the nucleoside reverse transcriptase inhibitors (NRTIs) abacavir (ABC), lamivudine (3TC), stavudine (d4T) and zidovudine (ZDV), the non-NRTIs efavirenz (EFV), etravirine (ETR) and nevirapine (NVP), and the integrase inhibitor raltegravir (RAL). Drug inhibition assays were performed using single-round HIV-1 entry assays with luciferase viruses pseudotyped with HIV-1 YU-2 envelope or vesicular stomatitis virus G protein (VSV-G). All the ARVs tested could effectively inhibit HIV-1 infection in macrophages, with EC_90_s below concentrations known to be achievable in the cerebral spinal fluid (CSF). Most of the ARVs had similar potency in astrocytes, however the NRTIs 3TC, d4T and ZDV had insufficient HIV-1 inhibitory activity in astrocytes, with EC_90_s 12-, 187- and 110-fold greater than achievable CSF concentrations, respectively. Our data suggest that 3TC, d4T and ZDV may not adequately target astrocyte infection *in vivo*, which has potential implications for their inclusion in Neuro-cART regimens.

## Introduction

Human immunodeficiency virus type 1 (HIV-1) penetrates the central nervous system (CNS) during acute infection or sometime shortly thereafter. Later in the course of HIV-1 disease it frequently causes encephalitis (HIVE), HIV-associated dementia (HAD) or less severe HIV-associated neurocognitive disorders (HAND) [1]. While it is the macrophage-lineage cells of the CNS that support productive HIV-1 replication, specifically the perivascular macrophages and microglia [1], [2], astrocytes undergo a restricted infection [3]–[5]. Nonetheless, recent studies have demonstrated that up to 19% of astrocytes can become infected in patients with the most severe HAD and HIVE, and therefore astrocytes potentially represent a significant viral reservoir [6]. Furthermore, whilst astrocyte infection is restricted, their infection results in cellular dysfunction. This is associated with changes in gene expression [7], loss of neuronal support, reduced ability to maintain glutamate levels and loss of integrity of the blood-brain barrier (BBB) [1], [4], likely contributing to the development of HAND.

Certain antiretroviral drugs (ARVs) have been shown to have potentially superior CNS efficacy, as determined by the CNS penetration-effectiveness (CPE) scoring system, which is based on available drug concentrations in the cerebrospinal fluid (CSF) [8], [9] and clinical data showing improved neurocognitive outcomes. These ARVs include the nucleoside reverse transcriptase inhibitors (NRTIs) abacavir (ABC), emtricitabine (FTC), lamivudine (3TC), stavudine (d4T) and zidovudine (ZDV), the non-NRTIs efavirenz (EFV), delavirdine (DLV), etravirine (ETR) and nevirapine (NVP), the protease inhibitors (PIs) indinavir (IDV), darunavir (DRV), lopinavir (LPV), the CCR5 antagonist maraviroc (MVC), and the integrase inhibitor raltegravir (RAL). Because of their favorable CNS penetration, these agents are used in so-called “neurologically active combined antiretroviral therapy”, or Neuro-cART regimens, with the premise that their superior bioavailability in the CNS would be optimal for treating CNS infection [10], [11]. However, the relative activity of CNS-penetrating ARVs in the different susceptible CNS cell types has yet to be determined. Furthermore, whether any or all of these agents have HIV-1 inhibitory activity in astrocytes at physiologically relevant concentrations is unknown.

In this study, we determined the HIV-1 inhibitory activity of the NRTIs ABC, 3TC, d4T and ZDV, the non-NRTIs EFV, ETR and NVP, and the integrase inhibitor RAL in cellular models of brain macrophage-lineage cells and astrocytes, with comparison to peripheral blood mononuclear cells (PBMC). Our results show that whilst all the ARVs tested have potent inhibitory activity in macrophages and PBMC at physiologically relevant concentrations, the NRTIs 3TC, d4T and ZDV have inadequate inhibitory activity in astrocytes, with 90% inhibitory concentrations (EC_90_) exceeding those achievable in the CSF. These results suggest that 3TC, d4T and ZDV may not effectively target astrocyte infection *in vivo*, which has potential implications for their inclusion in Neuro-cART regimens.

## Materials and Methods

### Antiretroviral Drugs

The characteristics of the ARVs used in the study are summarized in Table 1 and all were prepared as 10 mM stocks in DMSO except for RAL, which was prepared in water. The ARVs were chosen to predominantly represent those used in current Neuro-cART regimens [12], and consisted of NRTIs, NNRTIs and integrase inhibitors. While protease inhibitors are currently used in Neuro-cART, we were unable to analyze them in this study because of the single round virus assay used here and the nature of our luciferase readout.

**Table 1:** Biological and pharmacological properties of the ARVs.

ARV	Abbreviation	ARV Class^a^	Neuro-cART	ARV concentrations (**µ**M)				Highest non-toxic concentration of ARV (**µ**M)^f^
				Plasma^b^	CSF^c^	IC_50_ d	CPE^e^	
Abacavir	ABC	NRTI	Yes	5.2–10.9	0.5–1.8	0.2–1.5	3	45
Lamivudine	3TC	NRTI	Yes	4.4–8.7	0.05–1.1	0.8–4.9	2	60
Stavudine	d4T	NRTI	Yes	3.4–6.4	0.2–0.4	0.3–2.1	2	50
Zidovudine	ZDV	NRTI	Yes	4.5–6.7	0.1–0.4	0.01–0.04	4	50
								
Efavirenz	EFV	NNRTI	Yes	9.2–16.6	0.01–0.09	0.01–0.05	3	0.3
Etravirine	ETR	NNRTI	Yes	0.2–0.4	0.01–0.02	0.001–0.2	2	1
Nevirapine	NVP	NNRTI	Yes	7.5–16.9	1.3–10.9	0.02–0.1	4	10
								
Raltegravir	RAL	INI	Yes	0.1–4.5	0.01–0.3	0.002–0.01	3	1

a NRTI, Nucleoside reverse transcriptase inhibitor; NNRTI, Non-nucleoside reverse transcriptase inhibitor; INI, Integrase inhibitor.

b The *in vivo* plasma concentration range [36].

c The *in vivo* cerebrospinal fluid (CSF) concentration range [36].

d 50% inhibitory concentration range for HIV-1 [36].

e CPE, CNS penetration-effectiveness (The scale is from 1 to 4, with 4 being the most favorable CNS penetration-effectiveness) [12].

f Highest non-cytotoxic concentrations evaluated in a cell culture system in all cell types (SVG, PFA, PBMC, and MDM cells).

### Human ethics approval

Human fetal brain tissue and PBMC were obtained following informed written consent from all participants in the study. This has been respectively approved by the Human Ethics Committees from St Vincent's Hospital (HREC 08284) and from the University of New South Wales (UNSW Ethic approval HREC 03187).

### Cell lines and primary cells

The SVG astrocyte cell line [13] was cultured in Minimum Essential Medium (MEM) supplemented with 20% (vol/vol) heat-inactivated fetal calf serum (HI-FCS), 100 µg/ml of penicillin and streptomycin, and 2 mM of GlutaMAX (Invitrogen, USA). Primary human fetal astrocytes (PFA) were prepared as previously described [14], and were cultured in Dulbecco's Modified Eagle Medium (DMEM) supplemented with 10% (vol/vol) HI-FCS, 100 µg/ml of penicillin and streptomycin, and 2 mM of GlutaMAX (Invitrogen). PBMC were purified from the blood of healthy HIV-1-negative donors, stimulated with 5 µg/ml of phytohemagglutinin (Sigma, USA) for 3 days, and cultured in RPMI 1640 medium supplemented with 10% (vol/vol) HI-FCS, 100 µg/ml of penicillin and streptomycin, and 20 U/ml of interleukin-2 (Roche, Switzerland). Monocyte-derived macrophages (MDM) were produced from elutriated monocytes that were cultured for 5 days in RPMI 1640 medium supplemented with 10% (vol/vol) pooled human sera, 100 µg/ml of penicillin and streptomycin, and 12.5 ng/ml of macrophage colony-stimulating factor (M-CSF).

### Production and quantitation of Env-pseudotyped luciferase reporter viruses

Env-pseudotyped luciferase reporter viruses were produced by transfection of 293T cells with pCMVΔP1ΔenvpA, pHIV-1Luc, and either pcDNA3-VSVg or pSVIII-YU2 Env plasmids using Lipofectamine 2000 (Invitrogen) at a ratio of 1∶3∶1, as described previously [15]–[17]. Viruses pseudotyped with the CCR5-using HIV-1 YU-2 envelope glycoproteins were used for infections of PBMC and MDM, whereas SVG cells and PFA were infected with viruses pseudotyped with the vesicular stomatitis virus G protein (VSV-G) in order to achieve sufficient levels of viral entry for the inhibition assays. The supernatants containing virus pseudotypes were harvested 48 h later, filtered through 0.45 µm filters, titrated on each of the different cell types (TCID_50_ values were calculated), and stored at −80°C.

### Cell viability assay

ARV cytotoxicity was assessed in all cell types at 72 h post-drug exposure using the CellTitre-Glo Luminescent Cell Viability Assay (Promega, USA), according to the manufacturer's protocol.

### Virus inhibition assays

Assays were performed in all cell types in the presence of titrating concentrations of ARV. 5,000 SVG, 2,500 PFA, 200,000 PBMC, or 50,000 MDM cells/well were seeded into triplicate wells of 96-well plates. Twenty-four hours later, the culture medium was removed and replaced with medium containing the ARV or DMSO (0.5% vol/vol), and equivalent TCID_50_ infectious units of luciferase reporter virus were added to the cells. After a 16 h incubation at 37°C, the initial viral inoculum was removed and replaced with culture medium containing the same ARV or DMSO (0.5% vol/vol) concentrations. At 72 h post infection, the medium was aspirated, the cells were lysed and HIV-1 infection measured using the Luciferase Assay System (Promega) according to manufacturer's instructions. Luminescence was measured using a FLUOStar Optima microplate reader (BMG Labtech, Germany). Inhibition curves and the 50% (EC_50_) and 90% (EC_90_) effective concentrations were determined by nonlinear regression analysis as previously described [16]–[18], using GraphPad Prism software (version 5.0d; GraphPad Software, USA).

## Results

### Inhibitory activity of CNS-penetrating ARVs in CNS cell models

**Figure 1:**
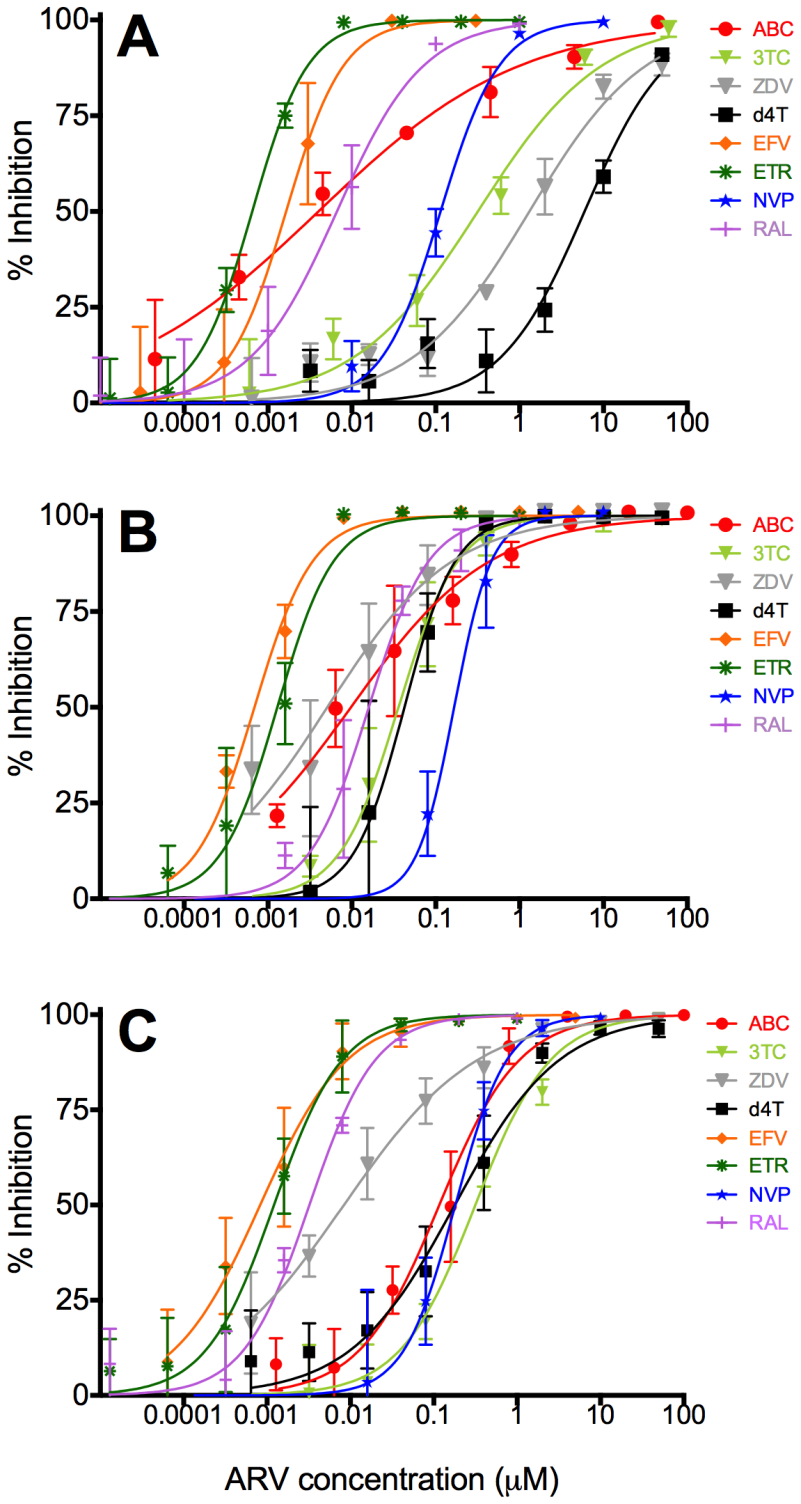
HIV-1 inhibitory activity of ARVs in primary cells. Virus inhibition assays were performed in PFA (A), MDM (B) and PBMC (C) using titrating amounts of ARVs, as described in Materials and Methods. The inhibition curves were generated as described in Materials and Methods, and were used to calculate the EC_50_ and EC_90_ values that are shown in Table 2 and Table 3, respectively. The data are a compilation of 4 independent experiments, each using cells obtained from separate independent donors, with each experiment conducted in triplicate. Shown are the means and SEM of these data.

We first evaluated the cellular cytotoxicity of the ARVs in the cell types studied. The characteristics of the ARVs, including their achievable plasma and CSF concentrations, inhibitory concentration ranges, CPE scores, and whether they have been used in Neuro-cART regimens are summarized in Table 1. None of the ARVs showed evidence of cytotoxicity at the highest concentrations tested in the virus inhibition assays (Table 1). Mitochondrial toxicity of the ARVs was not tested. Next, we evaluated the inhibitory activity of all the CNS-penetrating ARVs in cell models that represent brain astrocytes (the SVG astrocyte cell line and PFA), and brain macrophage-lineage cells (MDM), with comparison to that in a cell model that represents activity in the peripheral blood (PBMC). MDM were chosen as a model for brain macrophage-lineage cells because cell lines for perivascular macrophages do not exist and those for microglia are particularly poor at representing *in vivo* microglia. In addition, primary cells are extremely hard to obtain and difficult to culture and use. Our previous studies have shown that the HIV-1 replication characteristics in MDM recapitulate those observed in primary cultures of human fetal microglia [19], confirming their suitability as a convenient *in vitro* cellular model for macrophage-lineage cells *in vivo*. Virus inhibition curves are shown in Figure 1, and the EC_50_ and EC_90_ values are summarized in Table 2 and Table 3, respectively. Our results show that all the ARVs tested have inhibitory activity in SVG, PFA, MDM and PBMC, but with variable potencies (Tables 2 and 3). Of note, the EC_50_ and EC_90_ values for 3TC, d4T and ZDV were consistently higher in the SVG astrocytes and PFA compared to those in MDM and PBMC, suggesting reduced HIV-1 inhibitory activity by these particular NRTIs in astrocytes.

**Table 2:** ARV EC_50_ values for inhibition of HIV-1 in different cell types.

	EC_50_ (**µ**M) +/− SE^a^ (**µ**M)				
ARV	SVG	PFA	PBMC	MDM	
Abacavir	0.056±0.011	0.004±0.001	0.117±0.028	0.010±0.003	
Lamivudine	1.448±0.204	0.317±0.055	0.315±0.107	0.036±0.059	
Stavudine	0.578±0.189	5.963±0.622	0.179±0.061	0.042±0.046	
Zidovudine	0.017±0.003	1.311±0.130	0.008±0.002	0.005±0.002	
				
Efavirenz	0.002±0.000	0.002±0.000	0.001±0.000	0.001±0.000	
Etravirine	0.001±0.000	0.001±0.000	0.001±0.000	0.001±0.000	
Nevirapine	0.045±0.006	0.114±0.020	0.182±0.068	0.166±0.253	
				
Raltegravir	0.004±0.001	0.007±0.001	0.003±0.001	0.016±0.010	

a Mean 50% effective concentration±the standard error from n = 4 independent assays.

SVG, fetal astrocyte cell line; PFA, primary fetal astrocytes; PBMC, peripheral blood mononuclear cells; MDM, monocyte-derived macrophages.

**Table 3:** ARV EC_90_ values for inhibition of HIV-1 in different cell types.

	EC_90_ (**µ**M) +/− SE^a^ (**µ**M)				
ARV	SVG	PFA	PBMC	MDM	
Abacavir	0.369±0.055	1.738±0.354	1.318±0.141	0.636±0.084	
Lamivudine	43.942±4.805	13.451±2.315	3.101±0.471	0.214±0.156	
Stavudine	5.775±1.466	74.965±7.821	4.261±0.645	0.196±0.096	
Zidovudine	0.205±0.031	44.157±4.384	0.481±0.061	0.219±0.041	
				
Efavirenz	0.010±0.002	0.009±0.002	0.012±0.002	0.004±0.001	
Etravirine	0.003±0.001	0.003±0.000	0.010±0.001	0.007±0.001	
Nevirapine	0.543±0.054	0.669±0.114	0.867±0.145	0.508±0.345	
				
Raltegravir	1.072±0.162	0.091±0.016	0.024±0.003	0.096±0.027	

a Mean 90% effective concentration±the standard error from n = 4 independent assays.

SVG, fetal astrocyte cell line; PFA, primary fetal astrocytes; PBMC, peripheral blood mononuclear cells; MDM, monocyte-derived macrophages.

### The NRTIs d4T and ZDV have markedly reduced effectiveness in astrocytes compared to macrophages and PBMC

To better understand the apparent disparity between the inhibitory activities of 3TC, d4T and ZDV in astrocytes compared to the other cell models, we next undertook statistical analyses of the EC_50_ and EC_90_ values for these NRTIs in astrocytes compared to MDM and PBMC (Table 4). Here, we focussed on the results with PFA rather than SVG cells, as PFA represent the more relevant of the two cellular models for astrocytes *in vivo*. These analyses showed that the EC_50_ values for 3TC, d4T and ZDV in astrocytes were 8.8-, 142- and 262-fold greater than that in MDM, respectively (p<0.01). The EC_50_ values for d4T and ZDV in astrocytes were 33- and 164-fold greater than that in PBMC (p<0.01), respectively, but there was no difference for 3TC. The EC_90_ values for 3TC, d4T and ZDV in astrocytes were 63-, 382- and 202-fold greater than that in MDM, respectively (p<0.01). The EC_90_ values for d4T and ZDV in astrocytes were 18- and 92-fold greater than that in PBMC (p<0.01), respectively. The EC_90_ for 3TC in astrocytes was a modest 4.3-fold greater than that in PBMC, which neared significance. These results indicate that the NRTIs d4T and ZDV, and to a lesser extent 3TC, have reduced potency against HIV-1 in astrocytes compared to macrophages and PBMC *in vitro*.

**Table 4:** HIV-1 inhibitory activity of ARVs in PFA compared to MDM and PBMCs.

ARV	PFA compared to MDM				PFA compared to PBMC			
	EC_50_		EC_90_		EC_50_		EC_90_	
	Δ	*p*	Δ	*p*	Δ	*p*	Δ	*p*
Lamivudine	8.8	0.0079	63	0.0079	1.0	0.4524	4.3	0.0556
Stavudine	142	0.0079	382	0.0079	33	0.0079	18	0.0079
Zidovudine	262	0.0070	202	0.0048	164	0.0048	92	0.0095

Δ, fold change in EC_50_ or EC_90_ values.

P values (*p*) were determined using a non-parametric Mann Whitney U-test. Values <0.05 were considered statistically significant.

PFA, primary fetal astrocytes; PBMC, peripheral blood mononuclear cells; MDM, monocyte-derived macrophages.

### 3TC, d4T and ZDV EC_90_ values in astrocytes exceed those achieved in the CSF

**Figure 2:**
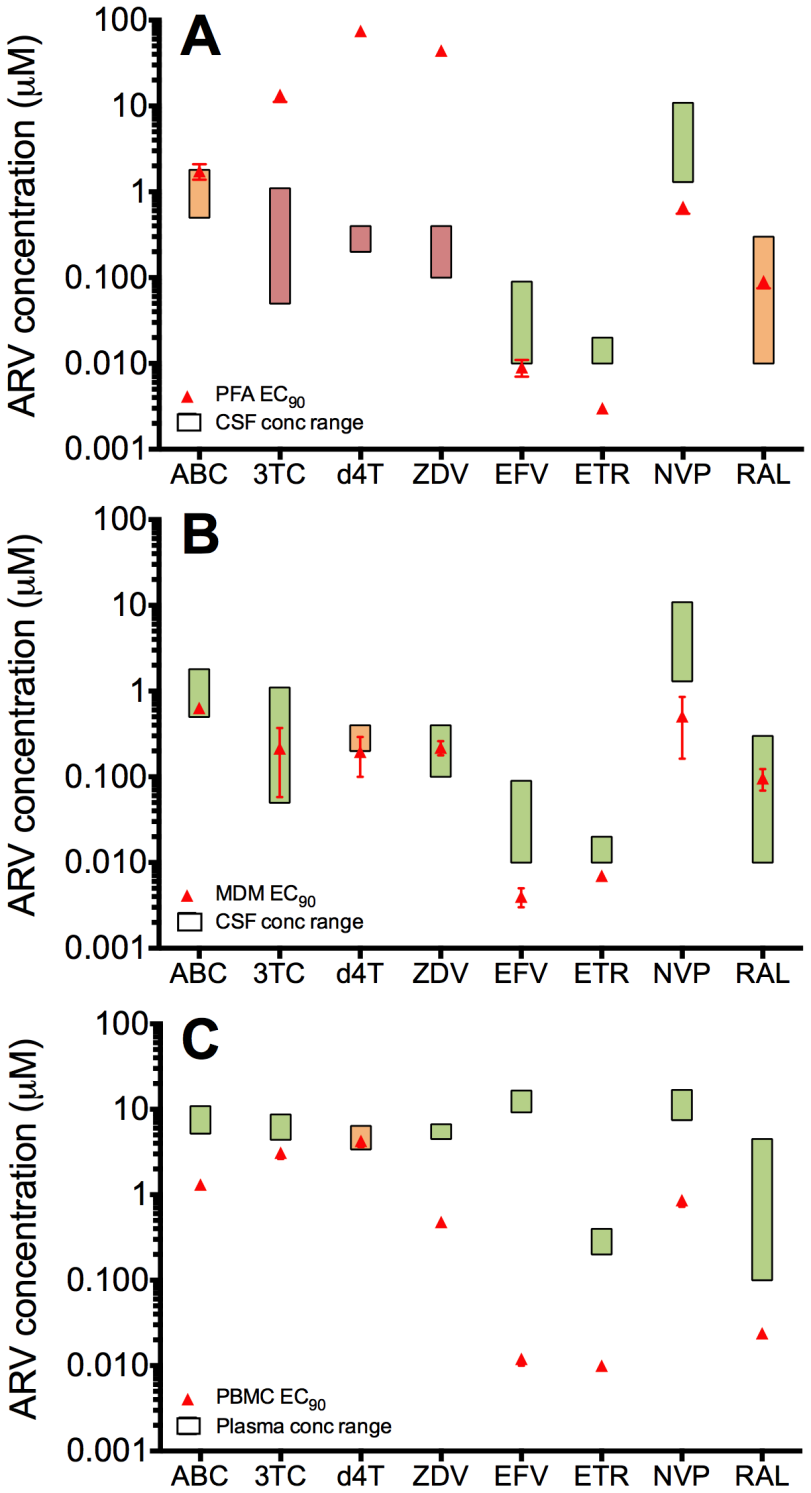
Comparison of ARV EC_90_ values in astrocytes, macrophages and PBMC to achievable *in vivo* concentrations. The ARV EC_90_ values determined in PFA, MDM and PBMC were compared to the relevant *in vivo* drug concentrations. PFA (A) and MDM (B) EC_90_ values were compared to the *in vivo* CSF drug concentration range, and PBMC EC_90_ values (C) were compared to the *in vivo* plasma drug concentration range. EC_90_ values are shown as red triangles with error bars representing the SE. Shaded boxes denote the relevant *in vivo* drug concentration range in either CSF (A and B) or plasma (C). The boxes shaded green indicate where the ARV EC_90_ falls below the physiological drug concentration range, the boxes shaded orange indicate where the ARV EC_90_ falls within the physiological drug concentration range, and the boxes shaded in red indicate where the ARV EC_90_ exceeds the physiological concentration range.

For an ARV to have effective inhibitory activity *in vivo*, its *in vitro* EC_90_ needs to fall below (or be equivalent to) the drug concentration that is achieved in the relevant tissue compartment [20], [21]. Therefore, although the preceding analyses showed that d4T and ZDV (and to a lesser degree 3TC) have reduced *in vitro* HIV-1 inhibitory activity in astrocytes compared to macrophages, the central issue that may have relevance to Neuro-cART regimens is whether their elevated EC_90_ values in astrocytes places these concentrations above what can be achieved by the drug in the CSF. We therefore next compared the EC_90_ values of each ARV obtained in the different cell models to the respective concentration range achievable in the CSF (for astrocytes and MDM) or plasma (for PBMC) (Figure 2). For these comparisons, the shaded boxes in Figure 2 indicate the physiological drug concentration ranges for each ARV in the CSF (Fig. 2A, B) and plasma (Fig. 2C), and the orange triangles represent the EC_90_ values of each ARV in astrocytes (Fig. 2A), macrophages (Fig. 2B) and PBMC (Fig. 2C). Here, the boxes shaded green indicate where the ARV EC_90_ falls below the physiological drug concentration range, the boxes shaded orange indicate where the ARV EC_90_ falls within the physiological drug concentration range, and the boxes shaded in red indicate where the ARV EC_90_ exceeds the physiological concentration range. These analyses show that the NRTIs 3TC, d4T and ZDV have EC_90_ values in astrocytes that exceed the achievable CSF drug concentrations (Fig. 2A). In contrast, the NRTI ABC, the NNRTIs EFV, ETR and NVP, and the integrase inhibitor RAL have EC_90_ values in astrocytes that are below or within the physiological concentration ranges in the CNS (Fig. 2A), and all the ARVs have EC_90_ values in macrophages (Fig. 2B) and PBMC (Fig. 2C) that are below or within the physiological concentration ranges in the CSF and plasma, respectively. These results suggest that there may be insufficient levels of 3TC, d4T and ZDV to inhibit HIV-1 infection of astrocytes *in vivo,* but that these drugs are likely to retain sufficient antiviral activity in the macrophage-lineage cellular compartments of the CNS.

## Discussion

Our results suggest that certain CNS penetrating ARVs used in Neuro-cART regimens may not be effective against HIV-1 infection of astrocytes. 3TC, ZDV and d4T are used in Neuro-cART regimens [8], [22], but here we show that the concentration of these drugs required to achieve virological suppression in astrocytes is 12-, 110, and 187-fold higher than what is achievable in the CSF, respectively. In contrast, 3TC, ZDV and d4T achieved virus inhibition in PBMC and MDM at concentrations that were below or within the respective concentration ranges that are achieved *in vivo*. Our results suggest that these NRTIs may not target all the susceptible HIV-1 target cell populations in the CNS, with potential implications for their inclusion in Neuro-cART regimens.

We observed some discordance in EC_50_ and EC_90_ values between the astrocyte cell line and primary fetal astrocytes. This highlights some of the limitations of using SVG for astrocyte work and reinforces the need to use the ‘gold standard’ PFA for confirmatory studies. In addition, while the EC_50_/EC_90_ values generated here are useful and insightful, they were generated in the context of a single round of infection. Therefore, further studies are required to analyze ARV effectiveness in the context of multiple rounds of infection.

Our recent studies showed that up to approximately 20% of astrocytes can be infected with HIV-1 *in vivo*, and therefore astrocytes represent a potentially significant reservoir of HIV-1 in the CNS [6]. Astrocytes are critical for maintaining normal brain homeostasis [23],_ENREF_17 and astrocyte dysfunction is known to contribute to HIV-1 neuropathogenesis [1], [4]. Continuing to use Neuro-cART regimens containing 3TC, d4T or ZDV could potentially lead to astrocyte infection remaining untargeted, which may contribute to neurocognitive impairment despite virological suppression in plasma. In support of this possibility, the prevalence of HAND is increasing despite suppressive cART [24]–[27].

The current focus on HIV-1 cure and eradication strategies has identified persistently infected viral reservoirs as a major barrier to the successful elimination of the virus [28]–[30]. These viral reservoirs are located within the brain, gut-associated lymphoid tissue, bone marrow, and genital tract [31]. HIV-1 infection of astrocytes is predominantly restricted to the expression of genes encoding the regulatory/accessory HIV-1 proteins [32], some of which are neurotoxic (for example the HIV-1 Tat protein) [33], and contributes to the persistent viral reservoir within the brain that is not cleared by the immune system or ARVs [29]. Confirming that the ARVs used in Neuro-cART regimens are active against-, and are present at concentrations sufficient for virological suppression in astrocytes may be important for controlling this potentially significant viral reservoir, and may be necessary to prevent the transcription of HIV-1 genes encoding neurotoxic viral proteins. The optimal targeting of HIV-1 infected astrocytes by ARVs may prevent the expansion of this HIV-1 infected cellular reservoir and potentially aid virus eradication efforts, and may also potentially contribute to better treatment outcomes by reducing neurotoxicity.

While we have shown that both ZDV and d4T have markedly reduced effectiveness in astrocytes compared to macrophages, the underlying mechanism for this remains unknown. All the NRTIs tested here require activation from their inactive native form by three sequential phosphorylation events. In the case of ZDV and d4T, the un-phosphorylated form is converted to the mono-, di- and tri-phosphorylated forms by the cellular thymidine kinases, thymidylate kinase and deoxynucleoside diphosphate kinase, respectively [34]. In contrast, 3TC (whose reduced HIV-1 inhibitory activity in astrocytes is relatively modest compared to that of d4T and ZDV) and ABC only have the final phosphorylation event in common, indicating unique cellular kinases are involved in their initial phosphorylation events. The drug is only active against the HIV-1 reverse transcriptase once the drug is tri-phosphorylated, by competing with the natural dNTP substrate for incorporation into DNA and causing chain termination. Three possible explanations could address the reduced effectiveness of d4T and ZDV in astrocytes; differences in cellular uptake of NRTIs, inefficient or incomplete drug activation (due to lower levels of cellular kinases or competition with the natural substrates for the kinases), and inefficient incorporation into DNA (due to higher levels of endogenous nucleotides). To this end, Perno *et al.* demonstrated that ZDV was more potent against HIV-1 infection in macrophage lineage cells due to lower levels of competing endogenous thymidine [35], which is a finding that is supported by our experiments when comparing EC_90_ values of ZDV between PBMC and MDM. Future work will explore this area to better understand the underlying cause of the markedly reduced effectiveness of the d4T and ZDV NRTI thymidine analogues in astrocytes.

In conclusion, the results of our study show that certain NRTIs, in particular the thymidine analogues ZDV and d4T (and to a lesser degree 3TC) have reduced HIV-1 inhibitory astrocytes *in vitro* compared to macrophage-lineage cells, and furthermore that the inhibitory concentrations of 3TC, ZDV and d4T in astrocytes exceed those achievable in the CSF. Thus, Neuro-cART regimens containing ZDV, d4T and/or 3TC may not effectively target HIV-1 infected astrocytes of the CNS. Furthermore, our results are the first to show differential ARV efficacy in brain cells, thereby introducing an important principle in the future analysis of ARVs.
